# Identification of Polymorphisms Associated with Drought Adaptation QTL in *Brassica napus* by Resequencing

**DOI:** 10.1534/g3.115.021279

**Published:** 2016-01-21

**Authors:** Richard S. Fletcher, David Herrmann, Jack L. Mullen, Qinfei Li, Daniel R. Schrider, Nicholas Price, Junjiang Lin, Kelsi Grogan, Andrew Kern, John K. McKay

**Affiliations:** * Cargill Specialty Seeds & Oils, Fort Collins, Colorado 80525; † Department of Bioagricultural Sciences and Pest Management, Colorado State University, Fort Collins, Colorado 80523; ‡ College of Agronomy and Biotechnology, Southwest University, Chongqing 400716, China; § Department of Genetics, Rutgers University, Piscataway, New Jersey 08854; ** Department of Computer Science, University of Toronto, Ontario M5S 2J7, Canada

**Keywords:** FLC, canola, flowering time, rapeseed, root pulling force (RPF)

## Abstract

*Brassica napus* is a globally important oilseed for which little is known about the genetics of drought adaptation. We previously mapped twelve quantitative trait loci (QTL) underlying drought-related traits in a biparental mapping population created from a cross between winter and spring *B. napus* cultivars. Here we resequence the genomes of the mapping population parents to identify genetic diversity across the genome and within QTL regions. We sequenced each parental cultivar on the Illumina HiSeq platform to a minimum depth of 23 × and performed a reference based assembly in order to describe the molecular variation differentiating them at the scale of the genome, QTL and gene. Genome-wide patterns of variation were characterized by an overall higher single nucleotide polymorphism (SNP) density in the A genome and a higher ratio of nonsynonymous to synonymous substitutions in the C genome. Nonsynonymous substitutions were used to categorize gene ontology terms differentiating the parent genomes along with a list of putative functional variants contained within each QTL. Marker assays were developed for several of the discovered polymorphisms within a pleiotropic QTL on chromosome A10. QTL analysis with the new, denser map showed the most associated marker to be that developed from an insertion/deletion polymorphism located in the candidate gene *Bna.FLC.A10*, and it was the only candidate within the QTL interval with observed polymorphism. Together, these results provide a glimpse of genome-wide variation differentiating annual and biennial *B. napus* ecotypes as well as a better understanding of the genetic basis of root and drought phenotypes.

*Brassica napus* is an amphidiploid formed from recursive and independent hybridizations between the diploid species *B. rapa* (A genome) and *B. oleracea* (C genome) (Nagaharu 1935; Parkin *et al.*1995). It is an important oilseed crop, with much of the global acreage considered “canola,” defined by grain which produces low quantities of erucic acid and glucosinolates. *B. napus* is produced commercially as both annual and biennial flowering ecotypes, which have been shown to represent genetically and morphologically distinct groups (Diers and Osborn 1994; Lühs *et al.* 2003). The genetic diversity differentiating these morphotypes represents a valuable source of variation for improving adaptation and ultimately increasing grain yields (Quijada *et al.* 2004, 2006). It will likely be an important resource in creating stress-adapted cultivars in both annual and biennial flowering types (Light *et al.* 2011; Rahman and Kebede 2011).

Plants cope with drought through physiological plasticity and/or adaptive mechanisms under heritable genetic controls (Juenger 2013). Drought coping strategies utilizing these mechanisms include drought escape, dehydration avoidance, and dehydration tolerance (Ludlow 1989). Dehydration tolerance is achieved through mechanisms that enable survival of low internal water potentials and is a strategy which exists in only 0.1% of angiosperm species (Oliver *et al.* 2010). Drought escape has been the focal strategy in breeding for low rainfall environments and focuses on rapid life cycling so that reproduction is completed prior to the onset of drought. Dehydration avoidance, in contrast, is the ability to maintain internal water status during drought conditions by minimizing water loss (*i.e.*, reducing transpiration) and/or maximizing water uptake (*i.e.*, larger root systems).

In a previous study, we mapped QTL for drought escape (flowering time), dehydration avoidance (root mass, measured by root pulling force), and grain yield in the SE-1 doubled haploid (DH) population of 225 lines grown under wet and dry treatments (Fletcher *et al.* 2015). The microspore donor plant used to create the DH mapping population originated from a cross between the cultivars IMC106RR and Wichita, which represent annual and biennial growth habits, respectively. The DH population exhibited large variation for all of the phenotypes analyzed resulting in the discovery of a total of 12 QTL for flowering time and root mass, some of which colocalized (Table 1). The QTL results provide valuable knowledge of the genetic architecture of these traits and the generally strong correlations among them. Yet, the causal polymorphisms remain unknown. One region of particular interest was the QTL located on chromosome A10 (*RPF.dry1/DTF.dry2*; hereafter referred to as *QTL.A10*). Conditional path analysis results suggest that it may impact both flowering time and root mass directly, indicating pleiotropy as the genetic mode of action. A deeper understanding of the genetic variation contained within *QTL.A10* is required for insight into the genetics underlying this and the other QTL regions identified in the SE-1 mapping population.

**Table 1:** Summary of QTL identified in Fletcher *et al.* (2015) with a significance threshold of *P* ≤ 0.05

Phenotype	Treatment	QTL	Linkage Group	Position (cM)	LOD	Variance Explained (%)
Days to flower	Wet	*DTF.wet1*	A02	15.4	4.01	2.21
		*DTF.wet2*	A03	102.0	8.11	4.67
		*DTF.wet3*	A10	75.0	37.69	30.05
		*DTF.wet4*	C02	65.0	41.43	34.50
	Dry	*DTF.dry1*	A03	104.2	4.19	3.92
		*DTF.dry2*	A10	75.0	24.19	28.97
		*DTF.dry3*	C02	66.0	33.58	45.51
Root pulling force	Wet	*RPF.wet1*	A10	73.6	13.73	18.46
		*RPF.wet2*	C02	63.3	14.78	20.09
		*RPF.wet3*	C07	95.0	3.34	4.02
	Dry	*RPF.dry1*	A10	76.0	12.15	19.71
		*RPF.dry2*	C02	63.3	7.22	11.12

QTL, quantitative trait loci; LOD, logarithm of odds.

Draft genomes of *B. napus* and its progenitors *B. rapa* and *B. oleracea* have become available recently (Wang *et al.* 2011; Cheng *et al.* 2011; Liu *et al.* 2014; http://www.ocri-genomics.org/bolbase/index.html, 2014; Chalhoub *et al.* 2014), prompting us to resequence the parent lines of our mapping population on the Illumina HiSeq (San Diego, CA) sequencing platform to investigate genetic variation contained within each QTL. An average of 30 gigabases (Gb, 2 × 100 bp reads) of sequence data from each parent was aligned to each available reference genome, allowing us to characterize genome-wide patterns of genetic variation differentiating the parent lines of the DH population. Gene ontology (GO) terms enriched for nonsynonymous substitutions were used to speculate on the major categorical differences among these divergent cultivars. In addition, discovered polymorphisms were used to characterize the extent of variation existing within QTL regions and to identify putative candidate genes contained therein, including *FLC* as the gene underlying *QTL.A10*.

## Materials and Methods

### Sequencing

This study analyzed the canola cultivars IMC106RR (Cargill Inc., National Registration No. 5118) and Wichita (Rife *et al.* 2001; Reg. no. CV-19, PI 612846) as well as a doubled haploid (DH) population of 225 lines derived from a cross between them as described in Fletcher *et al.* (2015). High quality DNA was extracted from the fifth leaf of each parent using the standard methods described in the Qiagen (Valencia, CA) column extraction kit. The extracted DNA was run on a 1% agarose gel to confirm DNA quality and concentrated to 50 ng/μl. Parental DNA libraries were sent to the University of Missouri DNA Core Facility (http://biotech.rnet.missouri.edu/dnacore/) and prepped for an average insert size of 200 bp. DNA libraries of each parent were sequenced on one lane of an Illumina HiSequation 2000 (San Diego, CA) sequencer to generate 2 × 100 paired-end reads.

### Alignment and polymorphism analysis

Mapping of the genomic sequencing data (fastq files) to the *B. napus* (Chalhoub *et al.* 2014), *B. rapa* (Wang *et al.* 2011; Cheng *et al.* 2011), and *B. olearacea* (Liu *et al.* 2014; http://www.ocri-genomics.org/bolbase/index.html, 2014) reference genomes was performed using SeqMan NGen v4 (DNAStar, Madison, WI). The alignment was performed using default settings for read mapping: k-mer size: 21; Minimum aligned length: 35; Maximum gap size: 6; Minimum match percentage: 93; Match score: 10; Mismatch penalty: 20; Gap penalty: 30; Alignment cutoff: 200. The mapping took into account the paired-end nature of the reads, required an insert size of 0–525 bp, and correct orientation in order to be designated as “properly paired” in the SAM/BAM output. The SNP calling parameters were as follows: Minimum SNP percentage: 5; Minimum probability SNP different than reference: 10%; Minimum SNP count: 2; Minimum base quality score: 5; Minimum strand coverage: 0; Bases to mask at end of reads: 0.

The SNP report created by SeqMan NGen was exported to ArrayStar v4 (DNAStar, Madison, WI) for further filtering. The final list of SNPs was generated using the following filter criteria: quality call score ≥ 30 (Phred scale), SNP frequency ≥ 5%, depth ≥ 5, and “p not ref” ≥ 90 (probability that the base is different than the reference base). Because SNPs were called relative to the reference genome, three lists were generated: SNPs differentiating both parents from the reference and SNPs present in only one of the parents (Trick *et al.* 2009). Thus, SNPs called in one parent and not the other were deemed to be polymorphic among parents. 500 randomly selected SNPs called across the genome of the *B. napus* alignment, along with 455 SNPs called in candidate genes of the *QTL.A10* interval, were manually reviewed using the Tablet (Milne *et al.* 2013) visual alignment tool to assess the results of the automated SNP caller. The results of these manual scores were used to estimate the SNP caller’s error rate which was then used in correcting genome-wide SNP calls. To be conservative, we did not include ‘hemi-SNPs’ (*i.e.*, polymorphisms originating from homologous reads, Trick *et al.* 2009) in our analyses as they may not represent genetic variants originating from the region of interest. Hemi-SNPs are the result of reads originating from a homologous locus that map to the incorrect locus due to a high degree of sequence homology. In the alignment they appear as heterozygous loci since two alleles are present for one parent but not the other (see Bancroft *et al.* 2011).

The Burrows-Wheeler Alignment (BWA) tool (Li and Durbin 2009) was used as part of a pipeline developed to estimate the number of loci to which each NGS read would map in the A and C genomes. First, fastq and reference genome data were input to BWA to generate read mapping information. Forward and reverse reads were mapped to the reference using a maximum edit distance of five. Since our reads were paired-end we used the sample option to generate alignments in the SAM format. In this part of the analysis we allowed for a maximum of 500 alignments (-n option) to be written in the XA tag. The SAM files output from this analysis were analyzed using a program written in Python to generate a data array containing the number of hits per read.

Polymorphisms were recorded as noncoding if they did not appear in a coding region of any gene model. SNPs in coding regions were then classified as either synonymous or nonsynonymous. The length of aligned sequence was calculated using the alignment summary report generated from SeqMan NGen. SNP density was calculated as the number of SNPs existing within a particular length of aligned sequence. In order to test Gene Ontology (GO) terms for an excess of nonsynonymous polymorphism, the number of nonsynonymous SNPs and the total number of codons in each gene model was obtained. For each GO term, the total density of nonsynonymous SNPs per codon among all gene models annotated with that term was calculated. Enrichment *P*-values were then calculated via permutation by randomly shuffling the numbers of nonsynonymous SNPs and codons across gene models while preserving GO annotations, and then asking whether the number of nonsynonymous SNPs per codon associated with the GO term in the permuted set was greater than or equal to the corresponding value from the true data set. 1000 such permutations were performed.

To estimate pairwise dN/dS ratios in IMC106RR and Wichita we incorporated the SNPs in the reference gene models and then, using the function CODEML implemented in the PAML package, we estimated dN/dS between each genotype and the reference. To estimate the 95% confidence intervals $\bar{dN}/\bar{dS}$ we used a paired bootstrap approach where we sampled (dN, dS) with replacement and calculated $\bar{dN}/\bar{dS}$ (10,000 bootstrap samples).

To test whether the divergence between the *B. napus* reference and each cultivar was significantly different from that of equal divergence, a Chi-square test was performed using the number of observed SNPs (Wichita: 355,048; IMC106: 789,793) relative to the expectation of equal divergence (572,420.5).

QTL genomic alignments were performed using the Mauve multiple genome alignment algorithm developed by Darling *et al.* (2004). The genomic sequence from each QTL was extracted from the relevant reference sequence. The “progressiveMauve” algorithm was employed to visualize the presence of large genomic rearrangements and/or inversions. Algorithm settings included: automatically calculate seed weight, automatically calculate the minimum LCB score, compute locally collinear blocks (LCBs), and perform a full alignment.

### Candidate gene identification and analysis

The LOD 1.5 confidence intervals of the genetic map positions of the QTL described in Fletcher *et al.* (2015) were determined in the R/qtl software package (Broman *et al.* 2003; Broman and Sen 2009). Markers spanning these intervals were compared with the *B. rapa* and *B. oleracea* references using BLAST (Altschul *et al.* 1990) to identify their physical map positions along with a list of genes expected to lie within them. Candidate genes were defined as those annotated with any term related to root and/or flowering.

### Sanger sequencing of FLC and FLC size polymorphism

Primers used to amplify the *FLC* homolog on A10 are given in Supporting Information, Table S8, as are additional sequencing primers. PCR amplicons were sequenced using BigDye Terminator v3.1 sequencing chemistry at the Colorado State University Proteomics and Metabolomics Facility. Primers spanning the insertion were used to score additional accessions (Table S7) for the FLC insertion based on simple size polymorphism of the PCR amplicon.

### Linkage analysis

KASP SNP genotyping assays (LGC Genomics, Teddington, Middlesex, UK) were developed for candidate SNPs discovered in comparison of the reference based assembly of the parents and used to genotype the original DH population. The parent genome alignments were used to design primer sequences for the KASP assays. A genetic linkage map was constructed which incorporated the molecular markers using JoinMap3 (Van Ooijen and Voorrips 2001) with a threshold recombination frequency of < 0.25 and a minimum logarithm of the odds ratio (LOD) score of 6 for creating linkage groups. Genetic distances were calculated using the Kosambi function (Kosambi 1944). Average genome-wide recombination rates for the A and C genomes were estimated by dividing the length of a particular genome’s physical map (bp) by that of its genetic map (cM).

Genome-wide QTL scans were performed using Haley-Knott Regression (Haley and Knott 1992) in R/qtl with 1 cM steps. QTL were selected using a step-wise model selection approach (Manichaikul *et al.* 2009) based on significance thresholds made from 1000 permutations (Churchill and Doerge 1994).

### Data availability

The raw sequence data used in this manuscript have been submitted to SRA (SRP065419).

## Results

### Resequencing of the B. napus genome

Whole-genome sequencing of IMC106RR and Wichita produced paired-end read data sets summing to 300 million reads (27.8 Gb passed Illumina filter) and 349 million reads (32.2 Gb passed Illumina filter), respectively. More than 90% of reads in each data set were considered to be of high quality (Q-score ≥ 30), equating to an average high quality read coverage of 26.6 × for Wichita and 23 × for IMC106RR. 85% of reads mapped to the ‘Darmor-*bzh*’ reference genome (Chalhoub *et al.* 2014), summing to nearly 532 Mb of callable (≥ 5 × coverage) sequence across the 645 Mb that constitute the 19 pseudomolecules of the assembly. Across the *B. rapa* (A genome; Wang *et al.* 2011; Cheng *et al.* 2011) and *B. oleracea* (C genome; Liu *et al.* 2014; http://www.ocri-genomics.org/bolbase/index.html, 2014) reference genomes, 82% of reads were mapped that summed to nearly 531 Mb of callable sequence aligning to the 642 Mb genome. The alignment made using the *B. napus* reference will hereafter be referred to as the “*B. napus* alignment” and the alignment made using the diploid progenitor references will hereafter be referred to as the “progenitor alignment.”

**Figure 1:**
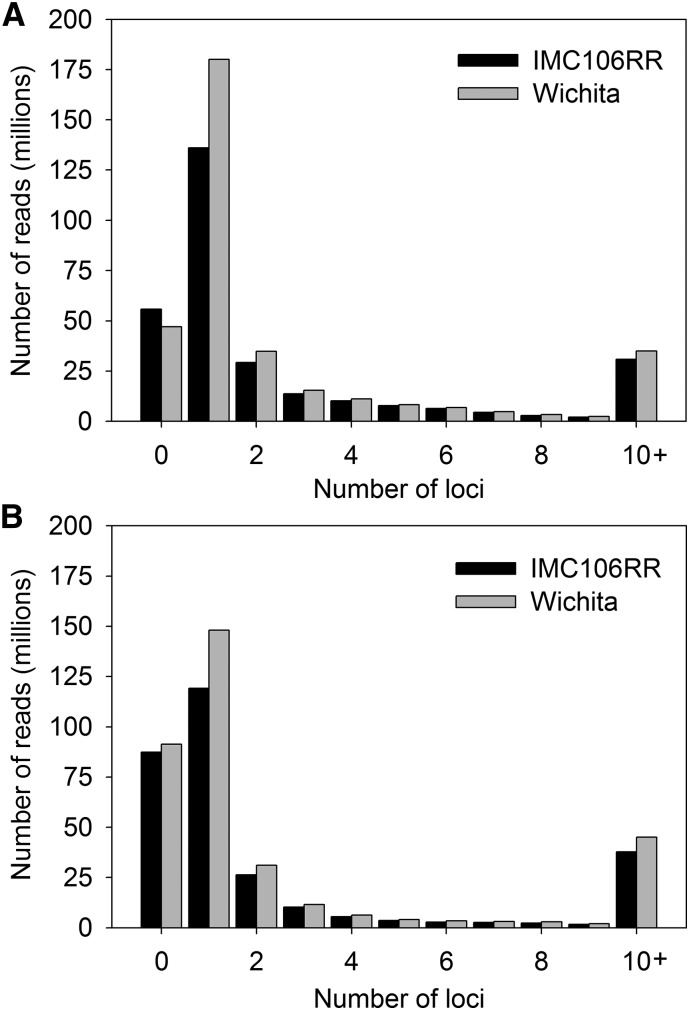
Histogram of the number of loci to which 100 bp Illumina HiSeq reads map. Reads for IMC106RR and Wichita, allowing 5% mismatch, mapped to (A) the *B. napus* (Chalhoub *et al.* 2014) and (B) the *B. rapa* (Wang *et al.* 2011; Cheng *et al.* 2011) and *B. oleracea* (Liu *et al.* 2014; http://www.ocri-genomics.org/bolbase/index.html) references.

*B. napus* is an amphidiploid that carries an average of six homologous regions due to the hypothesized hexaploid ancestry of both *B. rapa* and *B. oleracea* (Osborn *et al.* 1997; Cavell *et al.*1998; Parkin *et al.* 2005). Such genomic redundancy can affect alignments that rely on the single best match of short-reads. Therefore, we estimated the number of possible genomic regions that a single 100 bp read would map to each available reference at a minimum threshold of 95% sequence identity. The distribution of these analyses are characterized by a heavy right-skew where a diminishing number of reads map to many genomic regions (Figure 1). However, the median number of genomic regions to which a read maps is 1, suggesting that most reads originate from a unique region of the genome. Reads on the right tail of the distribution tended to be simple repetitive elements.

As an empirical measure of SNP call accuracy, we manually examined a subset of automated variant calls in the alignment visualization tool, Tablet (Milne *et al.* 2013). 500 SNPs were randomly selected across the *B. napus* alignment, of which 414 (82.8%) were confirmed to be accurate. A comparable accuracy rate (80.4%) was found in a similar examination of SNPs called in the progenitor alignment. The majority of the false positives were due to insufficient or no coverage in one parent, so that a well-supported SNP from the other parent appeared to be unique. Next, we searched each alignment for 100 SNPs randomly selected from the nearly 1200 used to construct the original linkage map. Of these, 81 were successfully identified in the *B. napus* alignment and 97 were identified in the progenitor alignment. The majority of the SNPs absent from the *B. napus* alignment blasted to the corresponding pseudochromosomes of the draft genome designated as “random.” These are a set of 19 pseudochromosomes constructed from scaffolds in which orientation could not be determined during assembly (see Supplementary Materials in Chalhoub *et al.* 2014). This suggests that additional genetic mapping data could enable accurate incorporation of these scaffolds to the existing pseudochromosomes, thus facilitating future QTL cloning efforts.

### Genome-wide SNP variation

Estimates of SNP density made using the progenitor and *B. napus* alignments were similar as IMC106RR and Wichita differed by an average of 0.21% (1.08 million SNPs) in the progenitor alignment and 0.23% (1.14 million SNPs) in the *B. napus* alignment (Table 2). SNP density varied substantially between homologous genomes and among chromosomes within them. T-tests comparing the average SNP density of the parent lines revealed a significantly higher rate (*P* ≤ 0.01) in the A genome than the C genome in both alignments (Table 2). We estimated the recombination rate within each genome, since it is hypothesized that mutation rate increases with higher recombination frequency (Gaut *et al.* 2007). Our results support this hypothesis as recombination occurred at a much higher frequency in the A genome (305 kbp cM^-1^) than in the C genome (513 kbp cM^-1^).

**Table 2:** Average SNP density estimates: comparisons of IMC106RR, Wichita, and each reference genome, by chromosome, based on the output of alignment to the progenitor genomes (utilizing the *B. rapa* and *B. oleracea* references) and the *B. napus* genome

	SNP Rate Comparisons (%)					
	IMC106RR–Reference		Wichita–Reference		IMC106RR–Wichita	
Chromosome	Progenitor	*B. napus*	Progenitor	*B. napus*	Progenitor	*B. napus*
A01	0.60	0.41	0.64	0.22	0.30	0.33
A02	0.59	0.24	0.61	0.22	0.17	0.17
A03	0.60	0.37	0.62	0.36	0.28	0.32
A04	0.62	0.32	0.64	0.27	0.26	0.29
A05	0.68	0.29	0.70	0.23	0.27	0.28
A06	0.65	0.17	0.67	0.07	0.22	0.21
A07	0.65	0.34	0.68	0.33	0.26	0.29
A08	0.67	0.31	0.69	0.16	0.26	0.26
A09	0.70	0.28	0.72	0.19	0.18	0.19
A10	0.61	0.40	0.64	0.13	0.30	0.31
A genome avg (s.d.)	0.64 (0.04)	0.31 (0.07)	0.66 (0.04)	0.22 (0.09)	0.25 (0.04)	0.26 (0.06)
C01	0.63	0.42	0.58	0.25	0.30	0.36
C02	0.62	0.38	0.71	0.27	0.27	0.32
C03	0.63	0.27	0.65	0.19	0.15	0.17
C04	0.59	0.22	0.59	0.11	0.18	0.17
C05	0.46	0.09	0.48	0.12	0.10	0.10
C06	0.42	0.25	0.45	0.16	0.15	0.19
C07	0.73	0.20	0.73	0.14	0.17	0.15
C08	0.59	0.16	0.61	0.06	0.13	0.13
C09	0.61	0.16	0.62	0.14	0.10	0.10
C genome avg	0.53 (0.09)	0.24 (0.10)	0.55 (0.09)	0.16 (0.07)	0.17 (0.09)	0.19 (0.09)
Genome-wide avg	0.60 (0.08)	0.28 (0.10)	0.62 (0.08)	0.19 (0.08)	0.21 (0.07)	0.23 (0.08)

SNP, single nucleotide polymorphism; Avg, average.

IMC106RR and Wichita were each more similar to the *B. napus* reference than they were to either of the diploid progenitor genomes (Table 2). A Chi-square test revealed that Wichita is more similar to the *B. napus* reference genome than IMC106RR (*P* < 0.0001). This result is perhaps not surprising given that Wichita and Darmor-*bzh* are both winter cultivars, and strong population structure differentiating winter and spring growth habit in *B. napus* is well established (Diers and Osborn 1994; Bus *et al.* 2011). However, this did not seem to affect the ability to call SNPs as the patterns of variation were highly congruent among alignments (r = 0.96; *P* < 0.0001). The results reported from this point onwards will be based on the progenitor alignment, since the outputs of each alignment are in general agreement (Table S1) and because the progenitor reference genomes have already been annotated to include orthologous *Arabidopsis* genes, thus enabling assignment of functional annotation to polymorphic gene sequences.

As expected, SNP density in coding regions was lower than noncoding regions in both the A and C subgenomes (Table 3). The synonymous substitution rate of 0.14% in the A genome was significantly higher than that observed in the C genome (0.08%; *P* < 0.01). However, the nonsynonymous substitution rate in each genome was nearly identical so that the ratio $\left(\bar{dN}/\bar{dS}\right)$ was elevated by an average of 0.15 in the C genomes of both IMC106RR and Wichita (Table S2). $\bar{dN}/\bar{dS}$ can be a metric for the selective pressure on coding loci where ratios less than one indicate purifying selection (Kimura and Ohta 1974). While purifying selection appears to be occurring in both genomes, our results suggest that it has been stronger in the A genome. Our estimates of differential recombination rates among the subgenomes seem to support this result as selection is expected to be more efficient with higher rates of recombination (Hill and Robertson 1966; Felsenstein 1974).

**Table 3:** Comparison of coding and noncoding SNP densities

Chromosome	Noncoding	Synonymous	Nonsynonymous	Total
A01	0.305	0.181	0.096	0.302
A02	0.178	0.089	0.055	0.173
A03	0.281	0.160	0.090	0.277
A04	0.259	0.145	0.085	0.256
A05	0.277	0.150	0.083	0.270
A06	0.225	0.129	0.073	0.222
A07	0.271	0.139	0.082	0.263
A08	0.272	0.116	0.077	0.259
A09	0.195	0.075	0.048	0.182
A10	0.304	0.185	0.098	0.303
A genome avg (s.d.)	0.257 (0.04)	0.144 (0.04)	0.080 (0.02)	0.251 (0.04)
C01	0.306	0.127	0.097	0.300
C02	0.727	0.158	0.119	0.272
C03	0.149	0.087	0.066	0.151
C04	0.186	0.076	0.066	0.183
C05	0.102	0.059	0.046	0.104
C06	0.151	0.071	0.062	0.151
C07	0.177	0.063	0.054	0.172
C08	0.130	0.057	0.051	0.129
C09	0.102	0.049	0.038	0.102
C genome avg	0.174 (0.07)	0.083 (0.04)	0.067 (0.03)	0.174 (0.07)
Genome-wide avg	0.218 (0.07)	0.115 (0.05)	0.075 (0.02)	0.214 (0.07)

Average estimates of noncoding, synonymous, nonsynonymous, and total SNP density between IMC106RR and Wichita across chromosomes of the A and C genomes. Avg, average; SNP, single nucleotide polymorphism.

Finally, we tested all genes carrying nonsynonymous variation for enrichment of particular GO terms in the hope of identifying particular classes that may broadly differentiate annual and biennial flowering types. A total of 25 GO terms were determined to be significant at *P* < 0.01 (Table S3). Of these, four were related to transcriptional regulation and the second lowest *P*-value was annotated as “response to gibberellin stimulus.” Both of these gene classes are well documented as being involved in the vernalization and flowering pathways so the enriched nonsynonymous variation in these GO terms may well describe some of the fundamental networks underlying differences in the growth habit of these cultivars (Zanewich and Rood 1995; Posé *et al.* 2012).

### SNP variation within QTL intervals

**Figure 2:**
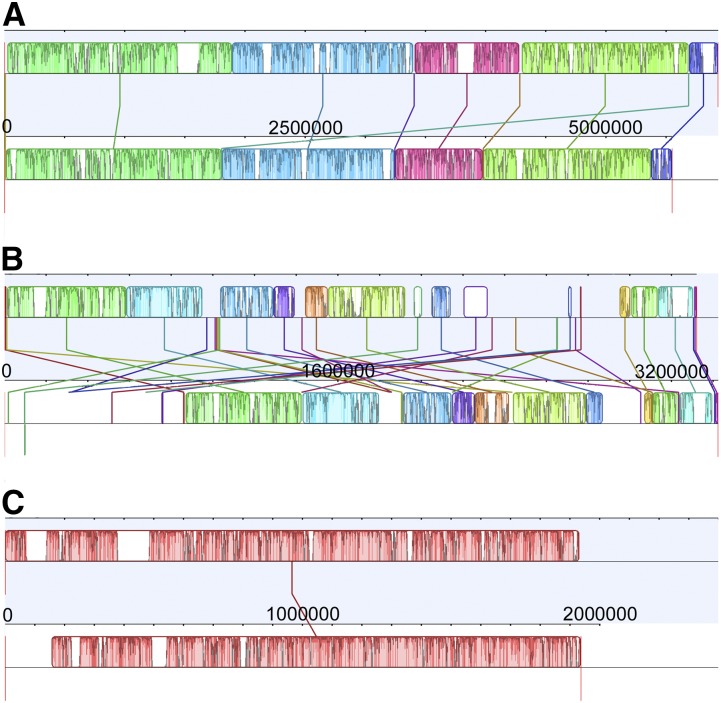
Alignment of quantitative trait locus (QTL) reference intervals. (A) Alignment of A02 QTL intervals from the *B. napus* (top; Chalhoub *et al.* 2014) and *B. rapa* (Wang *et al.* 2011; Cheng *et al.* 2011) references. (B) Alignment of C02 QTL intervals from the *B. napus* and *B. oleracea* (Liu *et al.* 2014; http://www.ocri-genomics.org/bolbase/index.html) references. (C) Alignment of A10 QTL intervals from the *B. napus* and *B. rapa* references.

We focused on differences in our five previously identified QTL intervals (Fletcher *et al.* 2015). 1464 genes were determined to carry at least one nonsynonymous substitution out of a total of 4373 genes predicted to lie within the five QTL intervals. Candidate genes with potential involvement in the control of each trait were selected from this list based on GO annotations related to roots and/or flowering (Table 4 and Table S4). Ultimately, the final list of candidate genes carrying nonsynonymous SNPs was narrowed down to 58 flowering genes and 19 root genes. Alignments comparing the progenitor and *B. napus* reference QTL demonstrate a noticeable difference in synteny among homologous genomes, where the A genome is more conserved than the C (Figure 2 and Figure S1). The disagreement is largely in the location of genes within each reference as the majority of genes are present in both QTL intervals, although in different positions.

**Table 4:** Number of genes carrying candidate polymorphisms in QTL regions

		No. Genes with Nonsynonymous SNPs / Total No. Genes		
QTL Chromosome	Trait	Total*^a^*	Flowering*^b^*	Root*^c^*
A02	Flowering	243 / 886	9 / 34	n/a
A03	Flowering	602 / 1762	28 / 52	n/a
A10	Both	212 / 624	8 / 25	3 / 20
C02	Both	263 / 440	13 / 33	13 / 25
C07	Root Mass	144 / 661	n/a	3 / 25
	Total	1464 / 4373	58 / 144	19 / 70

Summary of the number of genes carrying nonsynonymous SNPs relative to the total number of genes. SNP, single nucleotide polymorphism; QTL, quantitative trait loci; n/a, not applicable.

a Across the entire QTL.

b Annotated to flowering.

c Annotated to root function in QTL intervals of Fletcher *et al.* (2015).

We chose to conduct a more detailed analysis of *QTL.A10* to better understand the genetic factors potentially underlying the multiple traits that this QTL is controlling. Across the entirety of the *QTL.A10* interval, 17 genes were associated with roots, 22 were associated with flowering, and 3 were associated with both annotation categories (Table S5). The average SNP density across these 42 gene regions was 0.24% and ranged from 0–0.83%. Almost half of this initial list (19) showed no molecular variation and only 11 were deemed as candidate polymorphisms underlying QTL (*i.e.*, harboring one or more nonsynonymous mutations). Across these 11 genes, a total of 37 nonsynonymous polymorphisms were discovered. Bra009156 had a SNP density of 0.83%, the highest of any of the candidates analyzed. Finally, 12 consecutive candidate genes spanning from Bra008955 to Bra009327 showed no genetic variation, including the notable candidate gene *FLC*.

### QTL.A10 is characterized by a large insertion/deletion (indel) in FLC

**Figure 3:**
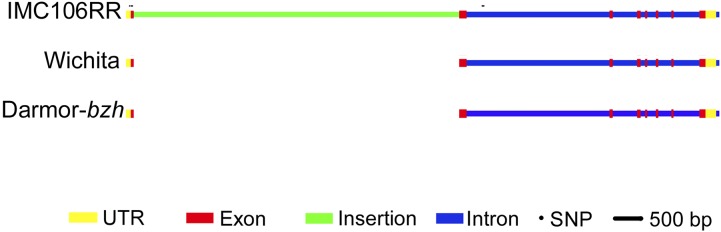
Diagram of *FLC* A10 homolog of Wichita and IMC106RR relative to the *B. napus* reference sequence from Darmor-*bzh*. In the first exon of BnaA10g22080D, IMC106RR has a 5624-base insertion not found in Wichita or the reference sequence. Scale bar, 500 bp. Circles indicate single nucleotide polymorphisms found relative to the reference sequence.

Despite the high degree of synteny across *QTL.A10* (Figure 2C) and lack of a SNP in *FLC* observed in the resequencing results, we examined *FLC* for sequence-length polymorphisms as these might not be captured with NGS methods. Using five DH lines from each allele class at *QTL.A10*, we found a large insertion of 5.6 kbp in the IMC106RR allele of *FLC* relative to Wichita. Sanger sequencing of the gene confirmed that the sequences of Wichita and IMC106RR were similar except for the insertion. Comparing our sequence to the gene models of *FLC* in *B. rapa* (Bra009055) and *B. napus* (BnaA10g22080D), the insertion was located in the first exon (Figure 3). The insertion sequence contains stop codons which are predicted to lead to a truncation in the protein. The insertion sequence also contains several conserved domains (Table 5), with an organization similar to long interspersed nuclear element (LINE) retrotransposons (Komatsu *et al.* 2003; Han 2010), including an endonuclease domain, reverse transcriptase, and ending with poly-A.

**Table 5:** Conserved domains in the *FLC* insertion sequence

Name	Accession	Description	Sequence Position	E-Value
DUF 4283	pfam14111	Domain of unknown function	780-1229	2.6 E-23
zf-CCHC_4	pfam14392	Zinc knuckle	1239-1385	1.3 E-05
exo_endo_phos	pfam03372	Endonuclease/exonuclease/phosphatase	2005-2664	2.8 E-15
RT_nLTR_like	cd01650	Non-LTR reverse transcriptase	3487-4278	1.2 E-57
RVT_1	pfam00078	Reverse transcriptase	3502-4278	7.1 E-46
zf-RVT	pfam13966	Zinc-binding in reverse transcriptase	5062-5319	6.4 E-30

LTR, long terminal repeat.

### Remapping in QTL.A10 suggests Bna.FLC.A10

**Figure 4:**
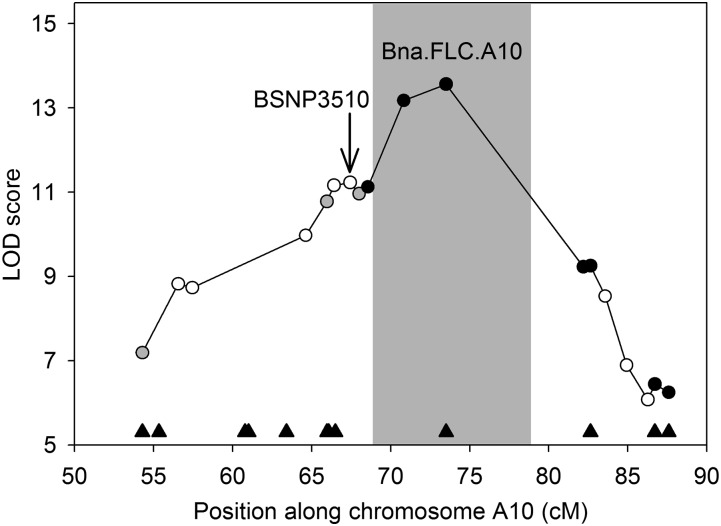
QTL remapping at *QTL.A10*. Logarithm of odds (LOD) score for the quantitative trait loci (QTL) at markers in the original QTL interval are given for the new mapping. Shading indicates the new confidence interval. White circles, original markers; gray circles, original markers and new markers located at similar genetic positions; black circles, new markers; black triangles, locations of candidate genes carrying nonsynonymous polymorphisms.

To confirm genomic locations, we developed a KASP marker assay (LGC Genomics, Teddington, Middlesex, UK) for a nonsynonymous SNP in every gene within the *QTL.A10* QTL that carried one. In addition, we developed assays for several loci within a region (72.7–83.5 cM) that lacked markers in our original map. Twelve loci passed the criteria for suitable primer and SNP marker development and were used to genotype the DH population. Nine SNP markers ended up passing the primer design criteria and segregated at the expected 1:1 ratio for each parental allele. We also genotyped the DH population for the *FLC* insertion. We reconstructed the genetic map and validated the location of these markers as all of them mapped to the predicted chromosome A10, and all recombination fractions were consistent with the order based on their physical locations (Figure 4). The original gap between 73.6 and 86.3 cM was reduced by 46% to a distance of 8.7 cM. Interestingly, the SNP located in Bra009167, which mapped to A10 as expected based on the *B. rapa* reference, is not predicted to carry an ortholog in the *B. napus* reference. This result, along with the reference genome QTL alignments (Figure 2), suggests that additional linkage map data will be helpful in improving the current draft genomes and understanding the extent of structural variation such as deletions and rearrangements.

As mentioned, *QTL.A10* was selected as the focal QTL for this study due to its impact on all measured traits, along with the possibility that it is directly involved in the control of root mass. This conclusion is based on QTL scans that determined this locus to be significant after conditioning upon flowering time, a genetically correlated trait (r_g_ = 0.48). Genome-wide QTL scans were reperformed using the new map to determine if the additional markers increased the QTL model fit parameters, relative to the original QTL results. In the original analyses, the most highly correlated SNP (BSNP3510) was located in the gene Bra008726 at position 13,586,650; however, the new QTL mapping places the most significant marker in *FLC* (Figure 4). Further, in the new QTL interval, *FLC* was the only remaining candidate gene with observed polymorphism (Figure 4). For all other traits colocalizing with this QTL, such as flowering time, model fit parameters were improved with the denser linkage map and the most highly correlated polymorphism was determined to be the indel in *Bna.FLC.A10* for four of the five traits (Table S6). RPF in the wet environment (*RPF.wet3*) was the only trait in which *Bna.FLC.A10* was not the nearest marker. Instead, it was the adjacent SNP located at 13,897,294 (near Bra009063), an upstream distance of only 41 kbp.

### Bna.FLC.A10 insertion is widespread in spring ecotypes

**Figure 5:**
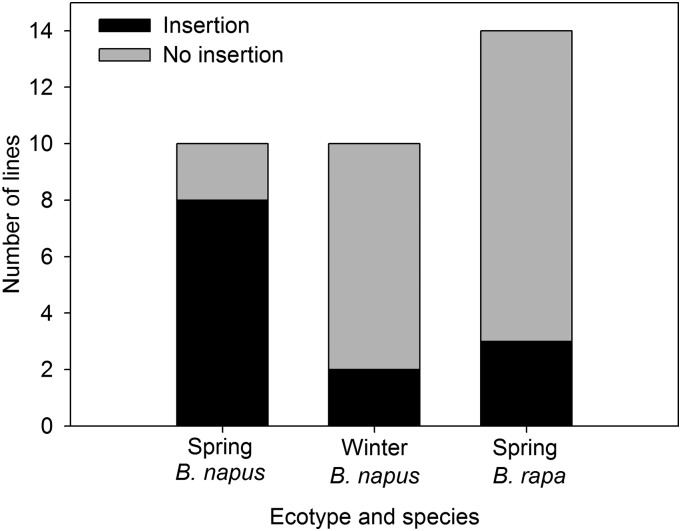
Prevalence of *FLC* insertion among ecotypes and species.

To test whether the *FLC* insertion is associated with flowering time and ecotype, we scored 20 spring and winter type lines for the insertion (Figure 5 and Table S7). There was a significant relationship between insertion presence and ecotype (*P* = 0.01), with the majority of spring types tested having the insertion. We also looked for the insertion in spring flowering lines of the parental species *B. rapa* to see if the origin of the insertion predates the allopolyploid speciation of *B. napus*. We did find the insertion in *B. rapa*, though at a lower frequency than in spring *B. napus* (Figure 5).

## Discussion

We previously mapped several QTL underlying drought-related traits in a DH population created from a cross between annual and biennial *B. napus* cultivars (Fletcher *et al.* 2015; Table 1). Here we utilize the draft genomes of three *Brassica* species to resequence the mapping population parent lines. The results revealed patterns of genome-wide molecular variation and identified major gene classes that may broadly underlie the different growth habits of the cultivars studied. In addition, candidate genes were identified at five QTL explaining variation in traits of importance to drought adaptation. In our focused analysis of *QTL.A10*, we developed molecular markers from a subset of the discovered SNPs and mapped them back onto the genetic map. QTL analyses using the new map identified *Bna.FLC.A10* as the most highly correlated polymorphism in the QTL region.

### Genome-wide variation differentiating the parent lines

This study utilizes the recently published draft genomes of *B. napus* and its diploid progenitors as references in resequencing *Brassica* genotypes. The results suggest advantages to utilizing all three reference genomes as they were comparable at the genomic-scale while differences became apparent at higher resolution. For instance, the SNP differentiating IMC106RR and Wichita identified in Bra009167 of the *B. rapa* reference genome mapped to the A10 linkage group of our genetic map. This is strong evidence that this gene is present at this same locus in both cultivars. However, Bra009167 is not predicted on A10 in the Darmor-*bzh* genome. Similar observations in other species suggest that presence/absence and other structural variants may be common within species, and thus a single reference genome is of limited utility (Tettelin *et al.* 2005; Morgante *et al.* 2007; Lu *et al.* 2015). Comparisons of the genomes of multiple maize cultivars found this type of structural variation to be common (Lai *et al.* 2010), suggesting that using several genomes that span the evolutionary history of the species as references will help mitigate these types of false negatives. Likewise, the candidate genes identified in our study cannot be considered comprehensive since they are only based on the *B. rapa* and *B. oleracea* reference genomes.

Our study provides a glimpse of genetic variation distinguishing the genetic pools of annual and biennial *B. napus* (Hasan *et al.* 2006; Bus *et al.* 2011). The A genome showed higher SNP diversity than the C genome, a result that is consistent with previous research (Trick *et al.* 2009; Durstewitz *et al.* 2010; Bancroft *et al.* 2011; Bus *et al.* 2012; Tollenaere *et al.* 2012; Delourme *et al.* 2013; Liu *et al.* 2014). Several of the previous studies included Asian cultivars that are known to have been bred with *B. rapa*. This breeding history is hypothesized to be the basis of the generally higher diversity observed repeatedly in the A genome (Qian *et al.* 2006). However, no Asian or *B. rapa* parent lines exist in the pedigrees of IMC106RR or Wichita, so previous results may be due to generally higher levels of diversity contributed from A genomes involved in the multiple independent allopolyploidy events that created modern *B. napus* (Song and Osborn 1992; Allender and King 2010). SNP diversity also varied greatly among chromosomes, particularly in the C genome. This may be an artifact of the severe bottleneck invoked by selection against erucic acid and glucosinolate accumulation during the creation of modern canola quality varieties (Downey and Röbbelen 1989; Sharpe and Lydiate 2003). QTL have repeatedly mapped to A09 and C09 for glucosinolate content in *Brassica* species (Howell *et al.* 2003; Feng *et al.* 2012), which are among the least divergent chromosomes in IMC106RR and Wichita (Table 2).

Contrary to the lower overall divergence discovered in the C genome, $\bar{dN}/\bar{dS}$ was significantly higher in the C genome (Table S2). This is indicative of a higher rate of purifying selection in the A genome (Kimura and Ohta 1974). This result is in agreement with the research of Zhao *et al.* (2013), where comparisons of molecular variation were made among paralogous genes of the progenitor *B. rapa* and *B. oleracea* genomes. Further, and again concordant with Zhao *et al.* (2013), we discovered a higher rate of recombination in the A genome. Recombination rate improves the efficacy of selection by creating haplotypes carrying multiple advantageous alleles and individuals with higher fitness (Hill and Robertson 1966; Felsenstein 1974). For the same reason, deleterious alleles are efficiently purged. The relaxed selection apparent in the C genome should be an expected result due to its lower rate of recombination. We might also consider the higher SNP density discovered in the A genome to be an expectation, since higher recombination rates seem to increase mutation rates in plants (Gaut *et al.* 2007). These patterns could also be the result of differences in effective population size (N_e_) of *B. rapa* and *B. oleracea* at the creation of *B. napus*. Increased N_e_ results in an improvement in the efficiency of selection so the patterns observed in this study could have been the result of elevated *B. rapa* N_e_ relative to *B. oleracea*. Of course, such a scenario would require many hybridization events among multiple genotypes of each species for patterns recognized at the level of the population to be captured in the allopolyploid descendants. The hybridization of the A and C genomes appears to have occurred on more than one occasion but the extent of its recursivity remains unclear (Palmer *et al.* 1983; Song and Osborn 1992; Song *et al.* 1988; Flannery *et al.* 2006; Allender and King 2010).

Gene annotations captured in GO terms provide a useful framework for identifying candidate genes and gene classes associated with particular phenotypes. We have identified a list of 25 GO terms associated with genes enriched for nonsynonymous substitutions. The GO terms in this list are likely to capture many of the genes broadly differentiating annual and biennial growth habit types. For instance, “response to gibberellin stimulus” was identified as an enriched term and its role in flower induction in biennial species is well established, where induction of flowering is possible through the application of gibberellic acid (Zeevaart 1983; Mutasa-Gӧttgens and Hedden 2009). Further, research in *Brassica* species has shown that vernalization increases GA biosynthesis and metabolism (Zanewich and Rood 1995), and delayed flowering in GA-deficient mutants (Rood *et al.* 1989).

### Candidate genes

Genome resequencing has proven to be an effective means of rapidly identifying candidate genes contained within QTL of *B. napus* (Tollenaere *et al.* 2012). In our study, we narrowed an initial list of 4373 genes contained across five QTL intervals down to 1464 genes carrying one or more nonsynonymous substitutions, which was narrowed further to a final list of 77 genes annotated to flowering or root (Table 4).

Flowering time has major impacts on fitness and yield under almost all conditions. An understanding of its genetic controls has implications for all facets of plant biology (Stinchcombe *et al.* 2004; Jung and Müller 2009). In our study, we have identified a total of 58 candidate flowering time genes across four QTL, and several of these represent strong candidates. Bra020249 and Bol008947 are located in the flowering time QTL on A02 and C02 and are orthologs of At5g60410 (a.k.a. *ATSIZ1*, *SIZ1*), a negative regulator of flowering (Jin *et al.* 2008). Of the 28 candidates located in the QTL interval on A03, the *Arabidopsis* orthologs of Bra001357 (At3g10390: *FLD*; He *et al.* 2006), Bra001729 (At3g18990: *VRN1*, *REM39*; Bastow *et al.* 2004), Bra013162 (At2g06210: *ELF8*, *VIP6*; Oh *et al.* 2004), Bra000392, and Bra000393 (At2g45650: *AGL6*; Yoo *et al.* 2011) all regulate the group of genes known as *FLC*/*MAF* that, in turn, impact flowering time significantly (Posé *et al.* 2012). Finally, the *Arabidopsis* ortholog of Bol036052 (At5g22290: *anac089*) contained within the QTL on C02 has been shown to have a direct role in the regulation of flowering time (Li *et al.* 2010). Ultimately, this study has provided a well-supported list of candidate flowering time genes for further research.

The root system plays a vital role in water and nutrient acquisition and, therefore, has an essential role in crop productivity (Sharp and Davies 1979). We have identified a total of 25 candidate genes involved in root development across three QTL of *B. napus*. Of the three candidates on A10, Bra009156 is noteworthy because the mutant phenotype of its *Arabidopsis* ortholog (At5g05980: *ATDFB*, *DFB*) is characterized by significant decreases in seedling root growth rate (Srivastava *et al.* 2011). We measured seedling root growth rate of 40 DH lines selected based on parental haplotype at this locus (20 lines representing each parent) using the method described in Mullen *et al.* (1998). A previous field study has shown that the parental haplotypes, as represented by these lines, differ in several aspects of root morphology including taproot dry mass, diameter, and length (Fletcher *et al.* 2015). There was no difference in seedling root growth rates of these 40 lines (*P* = 0.53), indicating that this locus most likely is not impacting root growth during this early phase of development. Of the list of 13 root related genes identified in the pleiotropic QTL on C02, Bol007157 (At5g18560: *PUCHI*) is an intriguing candidate due to its demonstrated role in both floral (Karim *et al.* 2009) and lateral root development (Hirota *et al.* 2007). None of the three genes identified in the QTL on C07 have a strong body of literature to support them as exceptional candidates. However, QTL have been repeatedly mapped to C07 for traits such as root length and mass (Hammond *et al.* 2009; Yang *et al.* 2010; Yang *et al.* 2011; Shi *et al.* 2012), suggesting that genes important to the evolution of root biology may be harbored on this chromosome.

### Remapping at QTL.A10

The resequencing data identified no molecular variation in the coding region of *FLC*, which was not surprising since most genetic variation in *FLC* exists in regulatory regions and introns (Gazzani *et al.* 2003; Yuan *et al.* 2009; Zou *et al.* 2012). Using sequence capture, Schiessl *et al.* (2014) found no mutation in *Bna.FLC.A10* in the early flowering line Campino, suggesting that this locus does not play a major role in flowering time regulation. In contrast, we found that Campino does have the insertion in *Bna.FLC.A10* (Table S7). We extended our search 200 bp beyond the putative 1 kb coding region upstream of *FLC* to look for the presence of a transposable element associated with the vernalization response in different *B. napus* genotypes, as reported by Hou *et al.* (2012), but found the region to be monomorphic. However, further sequencing identified a 5.6 kbp retroelement insertion in the first exon of *FLC* in the IMC106RR spring parent creating a truncated protein.

QTL scans showed the indel discovered in *Bna.FLC.A10* to be the most highly correlated polymorphism on the A10 linkage group. *FLC* is a MADS-box transcription factor that has been identified as an important regulator of the vernalization and autonomous flowering pathways in *Brassica* (Kole *et al.* 2001) due to its location within a network of genes (Michaels 2009). It has also been shown that the *FLC* protein binds to over 500 sites located throughout the *Arabidopsis* genome, and that those sites are enriched in gene ontology (GO) categories annotated with stress response and abiotic stimulus (Deng *et al.* 2011). Orthologous QTL regions encompassing *FLC* have been recurrently associated with flowering time QTL (Osborn *et al.* 1997; Schranz *et al.* 2002; Osborn and Lukens 2003; Long *et al.* 2007; Shi *et al.* 2009) as well as root QTL (Lou *et al.* 2007; Lu *et al.* 2008; Yang *et al.* 2010; Kubo *et al.* 2010) in the genus *Brassica*. Cumulatively, this leads us to hypothesize that the gene underlying these pleiotropic QTL may be *FLC*, but cannot conclusively exclude alternative gene(s) located within the *QTL.A10* interval. Here, we acknowledge that additional research (fine-mapping, transgenics, etc.) will be required to confirm the *Bna.FLC.A10* polymorphism as the causal factor of both the flowering time and root mass phenotypes. If true, the major challenge thereafter will be elucidating whether the pleiotropic action is the direct result of *Bna.FLC.A10* or simply a consequence of its upstream impact on flowering time (Fletcher *et al.* 2015).

## Supplementary Material

Supporting Information
