# Supplementary material for: Identification of Polymorphisms Associated with Drought Adaptation QTL in Brassica napus by Resequencing
Source: G3 (Bethesda). 2016 Jan 21;6(4):793–803. doi: 10.1534/g3.115.021279 (PMC4825650; doi:10.1534/g3.115.021279)
Supplement: Supporting Information [file supp_6_4_793__index.html]

Identification of Polymorphisms Associated with Drought Adaptation QTL in Brassica napus by Resequencing — Supporting Information 

# Identification of Polymorphisms Associated with Drought Adaptation QTL in *Brassica napus* by Resequencing

## Supporting Information for Fletcher *et al.*, 2016

**Files in this Data Supplement:**

- Figure S1 - Alignment of QTL intervals from the *B. napus* and progenitor references for the QTL not shown in Figure 2. (.pdf, 574 KB)
- Table S1 - List of primers for *Bna.FLC.A10*. (.xlsx, 12 KB)
- Table S2 - Average noncoding, synonymous, nonsynonymous, and total SNP density between IMC106RR and Wichita across chromosomes of the A and C genomes made using the *B. napus* (italics) and progenitor alignments. (.xlsx, 13 KB)
- Table S3 - Selective constraint ((dN) ⁄(dS) and 95% confidence intervals) estimated for the A and C genomes. (dN)⁄(dS) ratios were estimated using both IMC-reference and Wichita-reference comparisons. (.xlsx, 11 KB)
- Table S4 - Nonsynonymous SNP density and term enrichment P-Value of GO terms enriched for genes carrying nonsynonymous substitutions. (.xlsx, 12 KB)
- Table S5 - Full list of candidate genes contained within the five QTL intervals located on chromosomes A02, A03, A10, C02 and C07. (.xlsx, 16 KB)
- Table S6 - Summary of sequence coverage, polymorphism results and gene information for the 42 *Brassica rapa* candidate genes selected within the *QTL.A10* QTL. Genes in bold carried polymorphisms for which KASP assays were designed and used in mapping. (.xlsx, 18 KB)
- Table S7 - Comparison of A10 QTL model fit parameters generated using the original and NGS maps. LOD and R2 for days to flowering (DTF) in wet (1) and dry (2); root pulling force (RPF) in wet (3) and dry (4); and RPF conditional upon DTF (5) generated from genome-wide QTL scans using the original map (Fletcher *et al*. 2015) and NGS map (incorporating markers identified in this study). (.xlsx, 12 KB)
- Table S8 - Summary of insertion presence among spring and winter types. (.xlsx, 12 KB)
