# Supplementary material for: Identification of Polymorphisms Associated with Drought Adaptation QTL in Brassica napus by Resequencing
Source: G3 (Bethesda). 2016 Jan 21;6(4):793–803. doi: 10.1534/g3.115.021279 (PMC4825650; doi:10.1534/g3.115.021279)

Figure S1. Alignment of QTL intervals from the *B. napus* and progenitor references for the QTL not shown in Figure 2.

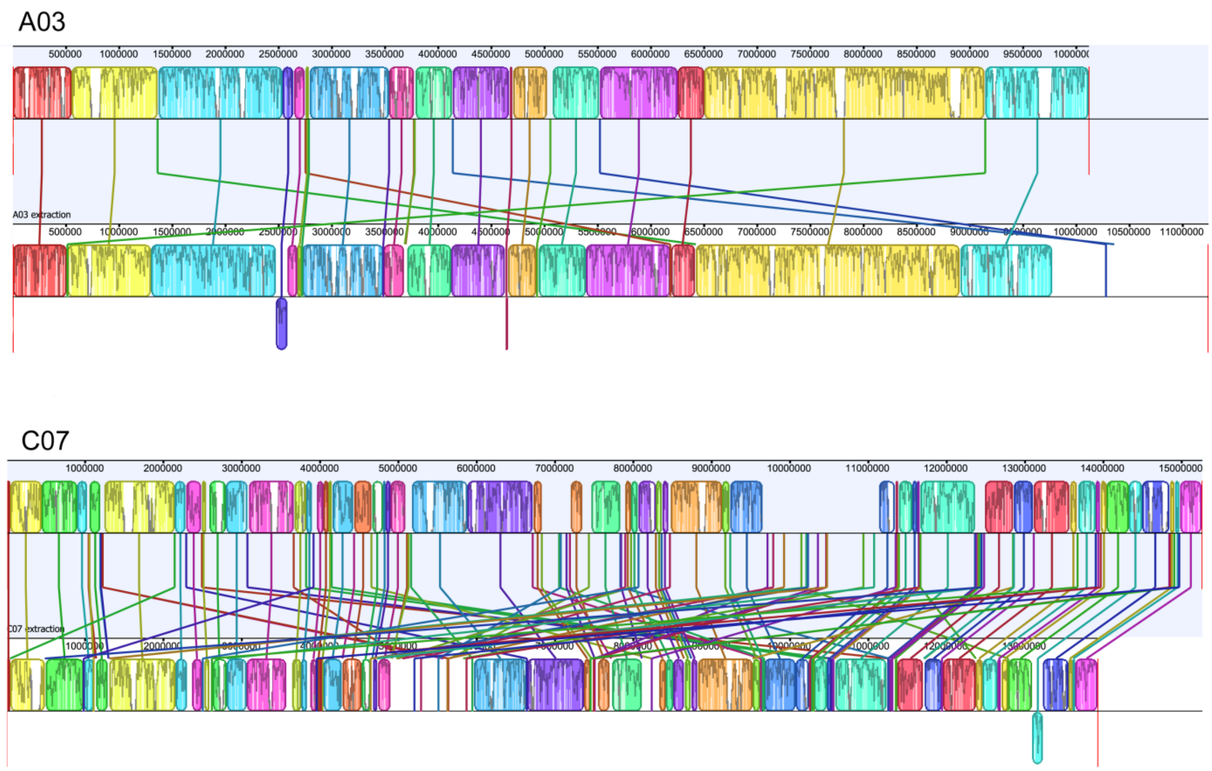

Supplement: Supporting Information [file supp_g3.115.021279_FigureS1.pdf]
